# Bioaccumulation of ultraviolet sunscreen compounds (mycosporine-like amino acids) by the heterotrophic freshwater ciliate *Bursaridium* living in alpine lakes

**DOI:** 10.1080/20442041.2017.1294348

**Published:** 2017-05-02

**Authors:** Bettina Sonntag, Barbara Kammerlander, Monika Summerer

**Affiliations:** ^a^ University of Innsbruck, Institute of Ecology, Innsbruck, Austria; ^b^ University of Innsbruck, Research Institute for Limnology, Mondsee, Austria

**Keywords:** *Bursaridium*, ciliates, high mountain lakes, MAAs, mycosporine-like amino acids, protists, ultraviolet radiation, *Urotricha*, UV photoprotection

## Abstract

Ciliates in shallow alpine lakes are exposed to high levels of incident solar ultraviolet radiation (UVR). We observed the presence of specific sunscreen compounds, the mycosporine-like amino acids (MAAs), in several populations of *Bursaridium*, a relatively large ciliate species found in such lakes. The populations from 3 highly UV transparent lakes revealed the presence of 7 MAAs (MG, SH, PR, PI, AS, US, and PE) in total concentrations of 3.6–52.4 10^−5^ μg μg^−1^ dry weight (DW) per individual, whereas in one glacially turbid and less UV transparent lake, no MAAs were detected in the *Bursaridium* population. The MAAs in the ciliates generally reflected the composition and relative amounts of the lakes’ seston MAAs, assuming that the ciliates fed on MAA-rich plankton. We experimentally found that naturally acquired MAAs prevented ciliate mortality under simulated UVR and photosynthetically active radiation (PAR) conditions. We further tested the dietary regulation of the MAAs-content in the ciliates under artificial UVR and PAR exposure and found an increase in MAAs concentrations in all treatments. Our assumption was that several stress factors other than irradiation were involved in the synthesis or up-regulation of MAAs.

## Introduction

In clear alpine lakes, the impact of solar ultraviolet radiation (UVR; 280–400 nm) is an essential factor influencing the life of planktonic communities. In the Alps, the ultraviolet-B radiation (UV-B; 280–315 nm) increases by ~19% per 1000 m altitude (Blumthaler et al. 1992). The potentially detrimental wavelengths of the UV-B and of the ultraviolet-A radiation (UV-A; 315–400 nm) completely pass the water column in these commonly shallow (*Z*_max_~15 m) and transparent lakes (Sommaruga and Psenner 1997). Aquatic plants and ambient vegetation in the lake catchment that may provide physical shelter from sunlight are usually absent. One factor responsible for high UVR transparency in lake water is the low amount of allochthonous chromophoric dissolved organic material (CDOM; Morris et al. 1995, Laurion et al. 2000). By contrast, glacier-fed lake water is highly turbid and absorbs UVR at the surface level, and, consequently, plankton is almost never exposed to high incident UVR (Rose et al. 2014, Kammerlander et al. 2016). Depending on the turbidity conditions, including more or less exposure to UVR, planktonic organisms are well adapted to varying levels of the incident solar radiation (Sommaruga 2001).

Microbial food webs of remote alpine lakes commonly harbor few morpho-species of bacteria, protists, and metazoans (Felip et al. 1999, Wille et al. 1999, Tartarotti and Sommaruga 2006, Kammerlander et al. 2016), although recent phylogenetic investigations revealed highly diverse protist communities in such habitats and strong biogeographic patterns (Traidó-Margarit and Casamayor 2012, Kammerlander et al. 2015, Filker et al. 2016).

Few studies, however, investigated how natural ciliate communities reacted to incident solar radiation (Wickham and Carstens 1998, Sommaruga et al. 1999, Sonntag et al. 2011a, 2011b, Moreau et al. 2014, Kammerlander et al. 2016). In one clear alpine lake, Kammerlander et al. (2016) identified the underwater solar UVR as the primary factor influencing the distribution pattern of the dominant ciliates prior to food resources (phytoplankton). UVR experiments conducted with ciliates identified species-specific effects resulting in, for example, DNA-damage, abnormal swimming behavior, retarded growth rates, or cell death (e.g., Giese 1945, Martini et al. 1997, Sanders et al. 2005, Summerer et al. 2009). Accordingly, ciliates use different photoprotective strategies under UVR stress, including physical shading by avoidance (e.g., seeking shelter in habitat areas where UVR is more strongly attenuated) or, for example, by the acquisition of sunscreen compounds. Which mechanism is applied by a specific ciliate species can so far be explained by their habitat preferences and UVR exposure history (Sonntag et al. 2011b, Slaveykova et al. 2016).

Photoprotection from incident UVR can be provided by the presence of a family of UV sunscreen compounds: the colorless and water-soluble mycosporine-like amino acids (MAAs) that absorb UVR between wavelengths of 309 and 362 nm (Shick and Dunlap 2002, Carreto and Carignan 2011). MAAs are found in a variety of marine and freshwater organisms including cyanobacteria, algae, rotifers, sea urchins, and algal-bearing ciliates (e.g., Caroll and Shick 1996, Banaszak et al. 2000, Tartarotti et al. 2004, Sommaruga et al. 2006, Sonntag et al. 2007, Obertegger et al. 2008, Summerer et al. 2008, Khanipour Roshan et al. 2015). For ciliates, several different MAAs were identified in the marine *Maristentor dinoferus* hosting zooxanthellae of the genus *Symbiodinium*, in *Stentor amethystinus* from Patagonian lakes, or in *Pelagodileptus trachelioides* from an Austrian lake (Tartarotti et al. 2004, Sommaruga et al. 2006, Sonntag et al. 2007). According to the prevailing incident solar radiation conditions in a lake, an ecophysiological regulation of the MAA content was suggested for *Askenasia chlorelligera* (Summerer et al. 2008). Until now, the origin of the sunscreen compounds has proved to be the symbiont in several freshwater mixotrophic species, even though evidence indicated that the ciliates’ diet was another source of MAAs (Sonntag et al. 2007, Summerer et al. 2008). To date, the biochemical pathways of MAAs synthesis have been described for algae, cyanobacteria, and bacteria, but they are unknown for ciliates (Balskus and Walsh 2010, Singh et al. 2010, Osborn et al. 2015).

Our hypothesis was that the algivorous heterotrophic ciliate *Bursaridium* may accumulate MAAs from phytoplankton (seston) that protect it against natural levels of UVR and photosynthetically active radiation (PAR). *Bursaridium* is a large planktonic ciliate ~150 μm in length commonly found in lakes located in the Central Alps (B.S. pers. observ.; Fried 1998). We tested several populations of *Bursaridium* from a set of alpine lakes for the presence and origin of MAAs. In 2 experimental setups, we (1) addressed the overall UV sensitivity of the ciliates under the assumption that they were well protected by naturally acquired MAAs, and (2) intended to prove a possible regulation of the MAA-content in the ciliate itself in a feeding experiment in which we offered algal food with and without MAAs.

## Study sites

We sampled the 3 UV transparent alpine lakes Hairlachersee (HAI), Oberer Plenderlesee (OPL), and Schwarzsee ob Sölden (SOS), and the glacier-fed turbid and less UV transparent lake Rifflsee (RIF). All 4 lakes are located within a radius of ~20 km (Table 1). HAI is only accessible by helicopter.

**Table 1.** Characteristics of the main study sites (from Laurion et al. 2002^1^ and present study data).

Lake and lake code	Coordinates	Altitude (m a.s.l.)	*Z*_max_ (m)	Optical appearance
Hairlachersee (HAI)	47°06′N, 10°51′E	2830	8.0	Clear
^1^Schwarzsee ob Sölden (SOS)	46°57′N, 10°56′E	2796	17.5	Clear
^1^Oberer Plenderlesee (OPL)	47°12′N, 11°02′E	2344	7.5	Clear
Rifflsee (RIF)	46°57′N, 10°50′E	2234	24.0	Glacier-fed turbid

## Methods

### Lake sampling

Abiotic conditions, optical properties, and chlorophyll *a* (Chl-*a*) were assessed on each sampling occasion for several years between 2002 and 2013. Water samples were taken at the deepest point of each lake from an inflated boat using a 5 L Schindler-Patalas sampler at depths of 0, 2, 4, and 6 m in HAI; 0, 3, 5, and 7 m in OPL; 0, 1.5, 3, 6, 9, 12, and 15 m in SOS; and 0, 1, 2, 4, 6, 10, 12, 14, 16, 18, and 20 m in RIF. Temperature was read directly from a thermometer attached inside the water sampler. Subsamples were collected to determine the water chemistry (1 L), Chl-*a* (1 L), seston MAAs (200 mL; HAI, RIF), and ciliate abundance (200–1000 mL; HAI only). Seston MAAs were determined for HAI and RIF only because values for SOS and OPL are available from literature (Laurion et al. 2002). Ciliate subsamples including triplicates were preserved with freshly prepared Bouin’s (5% final concentration) or acidified Lugol’s solution (once in 2012). Additionally, living ciliates were collected by vertical net hauls (10 μm gauze) for ciliate identification, MAAs analysis, and experimental procedures.

## *Abiotic parameters, Chlorophyll* a*, and optical properties*

In addition to temperature, standard limnochemical parameters were included in our study, comprising conductivity, pH, major ions, dissolved organic carbon (DOC), and nutrients (Table 2). Analyses followed the protocol of Sommaruga-Wögrath et al. (1997). Chl-*a* was used as a bulk parameter for assessing phytoplankton biomass; therefore, lake water was prefiltered (100 μm gauze), concentrated on glass-fiber filters (Whatman GF/F), and extracted with 90% acetone in the dark (24 h, 4 °C). After sonication (1 min, 35 W, Sonoplus, HD2070, Bandelin, Berlin, Germany), the extracts were cleared by filtration through glass-fiber filters (Whatman GF/F) and scanned in a spectrophotometer (Hitachi U-2001, Inula, Vienna, Austria) at 400–750 nm against an acetone reference. Chl-*a* concentrations were calculated following Lorenzen (1967).

**Table 2.** Abiotic parameters and nutrients measured during sampling (min, max) in Hairlachersee (HAI), Oberer Plenderlesee (OPL), Schwarzsee ob Sölden (SOS), and, Rifflsee (RIF). T = temperature, Cond = conductivity, SO_4_ = sulfate, Cl = chloride, Na = sodium, K = potassium, Mg = magnesium, Ca = calcium, NH_4_-N = ammonium, DN = dissolved nitrogen, TP = total phosphorus, DOC = dissolved organic carbon, DRSi = dissolved reactive silica, Chl-*a* = chlorophyll *a*, n.d. = not determined.

Lake	T (°C)	Cond (μS cm^−1^)	pH	SO_4_ (mg L^−1^)	Cl (mg L^−1^)	Na (mg L^−1^)	K (mg L^−1^)	Mg (mg L^−1^)	Ca (mg L^−1^)	NH_4_-N (μg L^−1^)	DN (μg L^−1^)	TP (μg L^−1^)	DOC (μg L^−1^)	DRSi (μg L^−1^)	Chl-*a* (μg L^−1^)
HAI	1.7–7.7	5.9–9.0	6.2–6.6	0.98–1.66	0.00–0.14	0.14–0.27	0.13–0.18	0.10–0.20	0.55–1.15	1–6	141–244	2.4–5.6	277–509	364–597	0.67–1.40
OPL	8.0–8.5	76.7–86.5	7.07–7.09	24.46–27.12	0.12–0.13	0.72–0.78	0.90–0.98	1.41–1.59	9.30–10.40	1–5	10–966	0.9–1.8	196–304	113–2714	n.d.
SOS	3.4–9.0	32.0–35.5	5.9–6.3	10.89–12.25	0.06–0.12	0.79–0.88	0.17–0.20	0.65–0.82	2.96–3.65	1–15	48–119	2.4–9.1	302–569	1085–1152	1.22–2.51
RIF	7.1–12.3	69.7–75.2	7.0–7.2	21.44–23.12	0.13–0.15	0.61–0.66	1.07–1.11	1.51–1.66	8.28–8.90	1–6	131–162	23.2–47.4	265–340	1214–1335	0.06–0.79

The incident downwelling irradiance at 305, 320, 340, and 380 nm and the downwelling PAR (400–700 nm) were measured with an *in situ* profiling UV radiometer (PUV-501B, Biospherical Instruments). The profiles were made within 2 h of solar noon. The diffuse attenuation coefficients (*K*_d_) of the downwelling UVR and PAR were calculated as the slope of an exponential regression between radiation and depth (*r*² > 0.99).

### Ciliate quantification and identification

Ciliates were identified from life observations as well as from specimens preserved and stained using the quantitative protargol staining method (Skibbe 1994, Pfister et al. 1999) following the identification keys of Foissner (1993) and Foissner et al. (1999). Living ciliates were observed with an Olympus SZ40 stereomicroscope and an Olympus BX50 microscope at magnifications of up to 1000× under differential interference contrast and brightfield illumination. Lugol’s preserved samples (also from the experiments) were counted in a Sedgewick Rafter Counting chamber with an inverted Nikon microscope.

## *Analyses of natural MAAs content of* Bursaridium

Starved individuals from HAI (2005, 2009–2012), SOS (2005, 2010, 2013), OPL (2004, 2009), and RIF (2010) were screened for the amount and composition of MAAs acquired in the ciliates cytoplasm. The extraction procedure and HPLC analysis followed the protocol of Sonntag et al. (2007). Because the concentrations of MAAs were normalized to the ciliates dry weight (DW), each individual collected for MAAs analysis was measured semi-automatically at 100× magnification using an Olympus BX50 microscope equipped with a CCD camera and a LUCIA image analysis software. The number of *Bursaridium* cells analyzed ranged from 5 to 76 individuals. Once, during a dinoflagellate bloom in OPL (2004), we extracted the MAAs of these dominant algae from a bulk sample to identify a possible MAAs source.

### Experimental approaches

Although we attempted to cultivate *Bursaridium*, all the numerous approaches failed (data not shown), and we therefore performed our experiments and analyses with individuals directly collected by hand from the original water samples. Before starting the MAAs extraction and experiments, the ciliates were cleaned individually over 5 drops of sterile filtered lake water. To ensure the complete digestion of ingested algae, they were kept in well plates overnight in a light/temperature controlled chamber at ambient lake water temperatures (5 °C) and a light/dark cycle of 16:8 h (80 μmol m^−2^ s^−1^ PAR, 0.10 W m^−2^ UV-A). Experiments were conducted within 1–2 days after lake sampling.

## *Sensitivity test of a natural* Bursaridium *population exposed under artificial UVR and PAR*

Under the assumption that they were well protected by their naturally acquired MAAs, starved individuals from HAI (2011, 2012) were placed into 12-well plates in sterile-filtered lake water and tested for their overall survival under simulated UVR+PAR (full spectrum), PAR only (exclusion of UVR with an Ultraphan-395 foil; UV-Opak, Digefra, Munich, Germany), and a dark control (well-plates wrapped in aluminum foil). Experiments were conducted in a walk-in climatic chamber equipped with 4 UV-A-340 fluorescent (8.60 W m^−2^ UV-A, 2.47 W m^−2^ UV-B; Q-Lab, Saarbrücken, Germany) and 2 visible fluorescence lamps (72 μmol m^−2^ s^−1^ PAR; Osram Cool White lamps NL-T8 36W/640-1, Vienna, Austria) at a constant temperature of 5 °C. The ciliates were exposed for 6 h to simulated natural irradiation conditions (weighted for the DNA Setlow action spectrum; Sommaruga et al. 1996).

## *Test for the dietary MAAs acquisition by* Bursaridium *under simulated UVR and PAR*

We tested the hypothesis that the dietary acquisition and regulation of MAAs by the ciliates was a response to the respective irradiation conditions. We expected that the ciliates accumulated MAAs from offered food and retained the dietary MAAs in their cytoplasm until the next day “in anticipation” of the next UVR and PAR dose. Therefore, individuals of *Bursaridium* from SOS were placed into 12-well plates filled with sterile-filtered lake water and fed with (1) an MAA-negative *Cryptomonas* sp. (strain 26/80, SAG culture collection of algae, Göttingen, Germany), (2) an MAA-positive *Peridinium inconspicuum* strain (SAMS Research services Ltd., UK), and (3) a mixture of both algae. *Peridinium inconspicuum* and cryptomonads are natural phytoplankton species found in SOS (Rott 1988), and bulk samples of both algae were collected for MAAs analysis. The experiment lasted for 2 days.

On day 1, we fed the ciliates with excess algae (21 000 ± 1600 *Cryptomonas* mL^−1^, 90 ± 30 *P. inconspicuum* mL^−1^) and exposed them under the experimental conditions and approaches discussed earlier. Under the stereomicroscope, after 6 h of exposure on day 1, all ciliates were individually cleaned from remaining algae in the medium as described, transferred to fresh medium without food, and kept in the dark overnight. On day 2, the same individuals were again exposed under the same irradiation and dark conditions without food and finally collected for MAAs analysis (7–12 individuals per approach). MAAs samples were stored at −80 °C until analysis (Tartarotti and Sommaruga 2002, Sonntag et al. 2007). Algae at T_0_ were counted from Lugol’s-fixed triplicate samples.

### Data analysis

To statistically test for differences in MAAs concentrations in the ciliates, Kruskal-Wallis one-way analyses of variance (ANOVA) were performed. Spearman rank order correlations were conducted among ciliates abundance, abiotic lake parameters, seston MAAs, and Chl-*a* (HAI only).

## Results

## *Abiotic parameters, Chlorophyll* a*, and optical properties*

Water temperatures were 1.7–9.0 °C in the clear lakes and 7.1–12.3 °C in RIF; conductivity was lowest (5.9–9.0 μS cm^−1^) in HAI and 32.0–86.5 μS cm^−1^ in the other 3 lakes; pH in HAI and SOS was 5.9–6.6 and around neutral in OPL and RIF (7.0–7.2); sulfate was lowest in HAI (0.98–1.66 mg L^−1^) and highest in OPL (24.46–27.12 mg L^−1^); chloride was 0.0–0.15 mg L^−1^ in the 4 lakes; sodium was 0.14–0.27 mg L^−1^ in HAI and 0.61–0.88 mg L^−1^ in the other 3 lakes; potassium was lowest in HAI and SOS (0.13–0.20 mg L^−1^) and 0.90–1.11 mg L^−1^ in OPL and RIF; magnesium was lowest in HAI (0.10–0.20 mg L^−1^) and 0.65–1.66 mg L^−1^ in the other 3 lakes; calcium was lowest in HAI and SOS (0.55–3.65 mg L^−1^) and 8.28–10.40 mg L^−1^ in OPL and RIF; ammonium was 1–6 μg L^−1^ in HAI, OPL, and RIF, and the max was detected in SOS (1–15 μg L^−1^); min and max dissolved nitrogen were detected in OPL (10–966 μg L^−1^); total phosphorus was low in the clear lakes (0.9–9.1 μg L^−1^) and 23.2–47.4 μg L^−1^ in RIF; DOC was low in all lakes, (196–569 μg L^−1^); min and max dissolved reactive silica were found in OPL (113–2714 μg L^−1^); Chl-*a* was 0.67–1.40 μg L^−1^ in HAI, 1.22–2.51 μg L^−1^ in SOS, and lowest in RIF with 0.06–0.79 μg L^−1^ (Table 2). Differences in the optical appearance among the lakes are reflected by the *K*_d_ of UVR and PAR (Tables 1 and 3).

**Table 3.** Diffuse attenuation coefficients (*K*_d_) at 305, 320, 340, and 380 nm wavelengths and for photosynthetically active radiation (PAR) in Hairlachersee (HAI), Oberer Plenderlesee (OPL), Schwarzsee ob Sölden (SOS), and, Rifflsee (RIF). Chl-*a* = specific seston MAAs in μg [μg Chl-*a*]^−1^ and the relative contribution of the different seston MAAs in % from HAI and RIF (study measurements) and from Laurion et al. (2002)^1^ for OPL and SOS. MG = mycosporine-glycine, SH = shinorine, PR= porphyra-334, PI = palythine, AS = asterina-330, US = usujirene, PE = palythene, n.d. = not determined.

Lake	Date	*K*_*d*_ 305 (m^−1^)	*K*_*d*_ 320 (m^−1^)	*K*_*d*_ 340 (m^−1^)	*K*_*d*_ 380 (m^−1^)	*K*_*d*_ PAR (m^−1^)	Chl-*a*–specific seston MAAs (μg [μg Chl-*a*]^−1^)	MG (%)	SH (%)	PR (%)	PI (%)	AS (%)	US (^1^MAA-357) (%)	PE (%)
HAI	21 Sep 2011	0.54	0.48	0.43	0.25	0.25	n.d.	n.d.	n.d.	n.d.	n.d.	n.d.	n.d.	n.d.
	30 Sep 2010	n.d.	n.d.	n.d.	n.d.	n.d.	20.0 ± 4.5	traces	86 ± 0	traces	1±0	13 ± 0	traces	traces
OPL	28 Jul 2004	0.29	0.24	0.17	0.12	0.15	n.d.	n.d.	n.d.	n.d.	n.d.	n.d.	n.d.	n.d.
	^1^21 Jul, 10 Oct 1998	n.d.	n.d.	n.d.	n.d.	n.d.	0.12–3.65	—	87–93	—	3-7	traces–10	—	—
SOS	5 Sep 2005	0.43	0.39	0.34	0.24	0.18	n.d.	n.d.	n.d.	n.d.	n.d.	n.d.	n.d.	n.d.
	^1^30 Jul 1998	n.d.	n.d.	n.d.	n.d.	n.d.	3.45	traces	71	—	22	traces	7	traces
RIF	23 Aug 2010	n.d.	n.d.	n.d.	n.d.	n.d.	0.13 ± 0.1	—	49 ± 33	18 ± 13	1 ± 2	—	5 ± 12	—

### Ciliate identification and quantification

**Figure 1.**
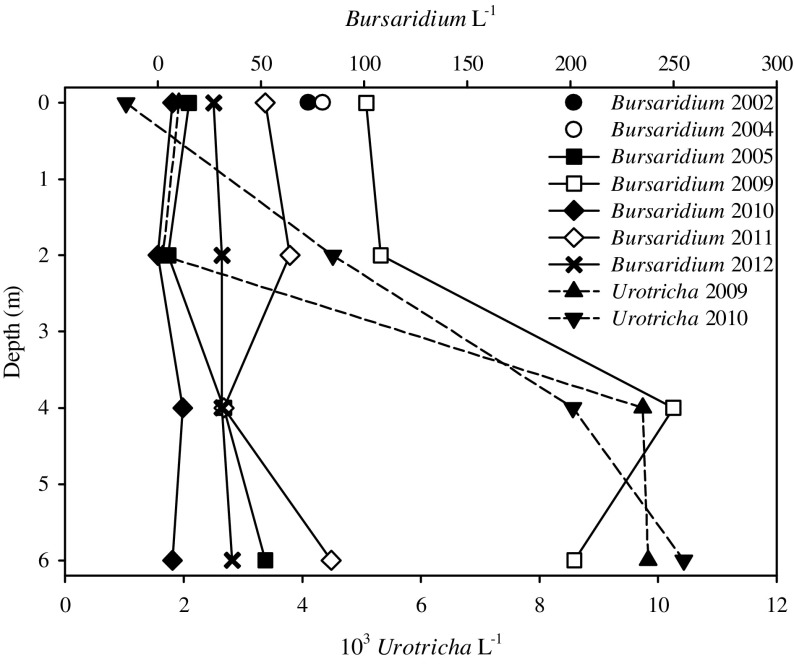
Vertical distribution of *Bursaridium* sp. (2002–2012) and *Urotricha* sp. (2009 and 2010) in Hairlachersee. In 2002 only one surface sample was taken, and in 2004 the lake was already ice-covered, and one sample was taken from directly under the ice.

Identifications of the 4 *Bursaridium* populations revealed that they belong to the same as-yet undescribed morpho-species (B.S. description in preparation). In HAI, another as-yet undescribed *Urotricha* species was identified. Numbers of *Bursaridium* were low (~50 ind. L^−1^ on average over all sampling dates), and the abundance of the *Urotricha* (counted in 2009 and 2010 only) were high (~6000 ind. L^−1^; Fig. 1). Temperature profiles in HAI (data not shown) revealed lake mixis, reflected in the ciliates’ vertical distribution except for 2009. Overall, no significant correlations were found among any pair of variables tested (*P* > 0.050).

**Figure 2.**
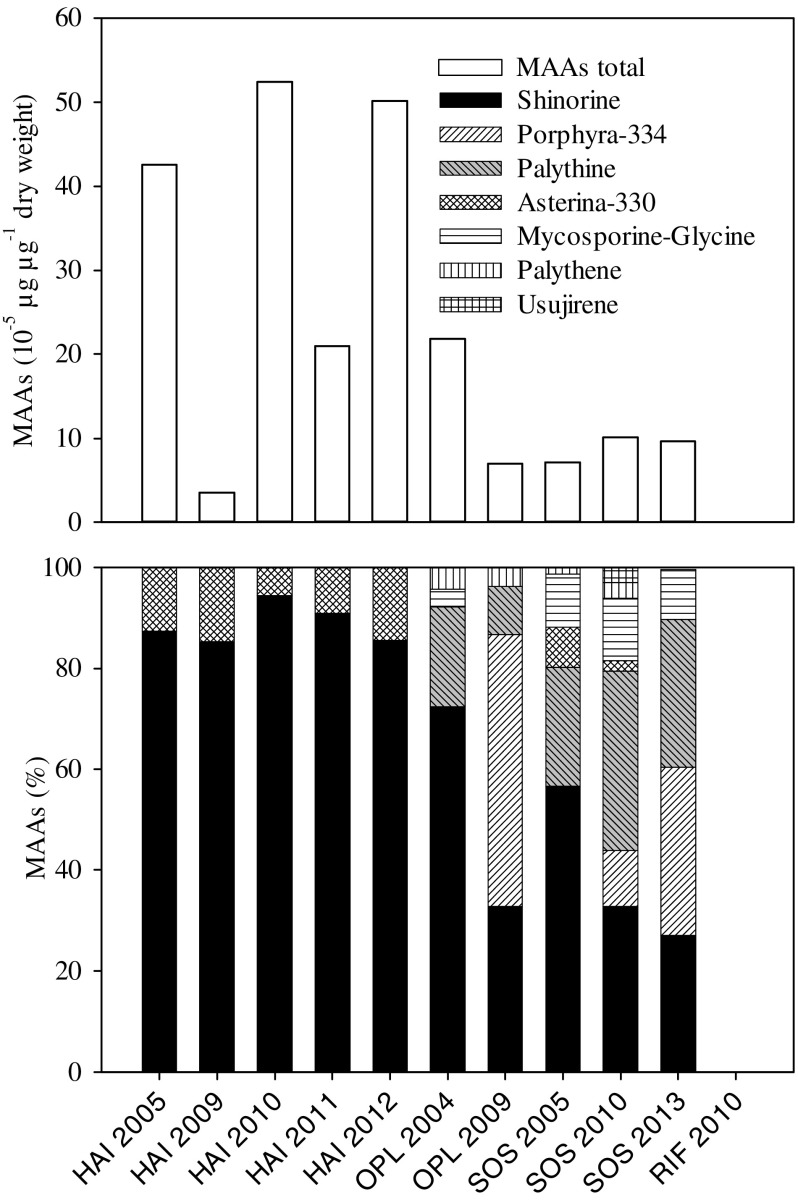
Natural mycosporine-like amino acids (MAAs) concentrations in starved *Bursaridium* populations from the alpine lakes Hairlachersee (HAI), Oberer Plenderlesee (OPL), Schwarzsee ob Sölden (SOS), and Rifflsee (RIF). Total MAAs concentrations (10^−5^ μg μg^−1^ DW, upper panel) and their relative contribution (%, lower panel) are presented.

**Figure 3.**
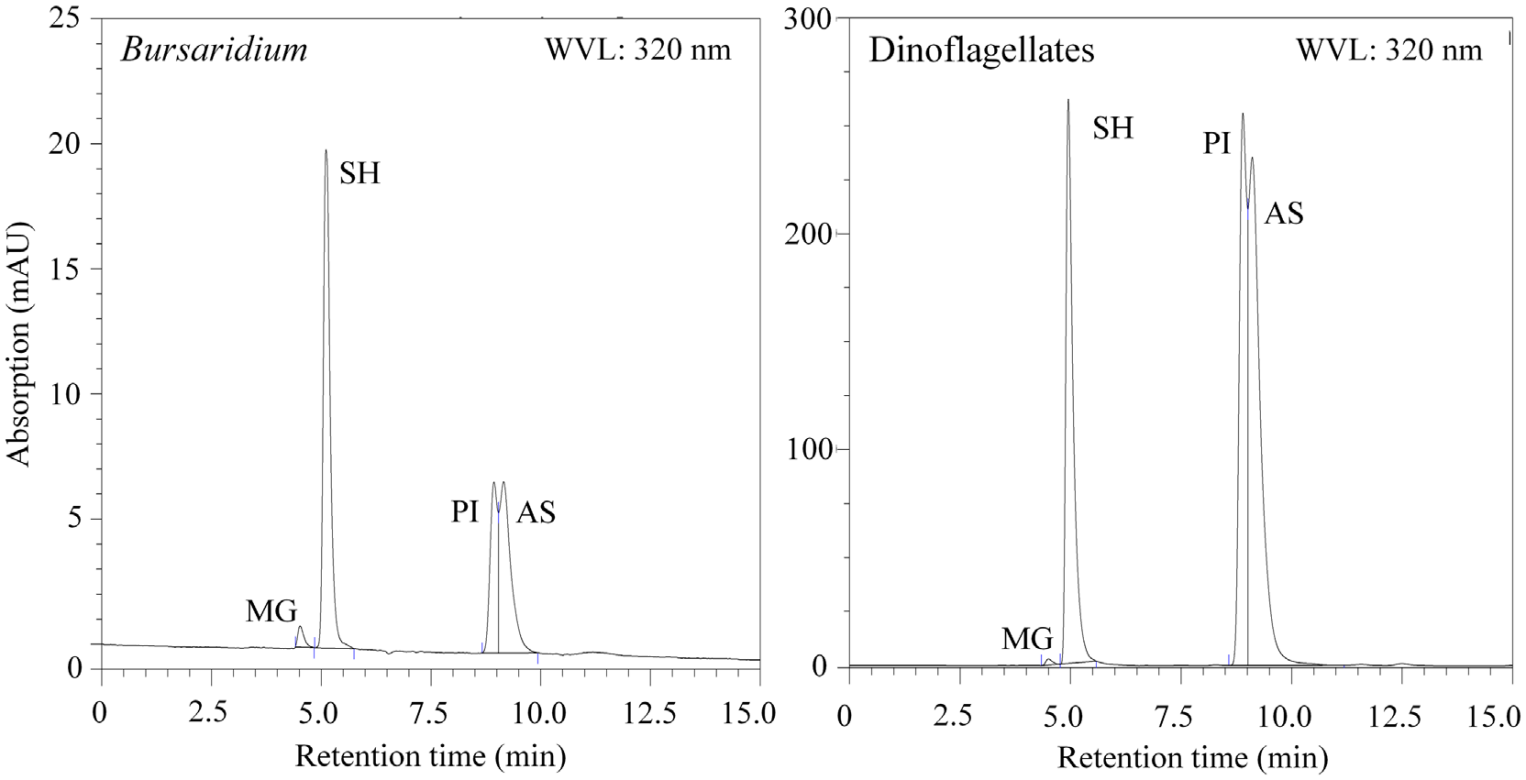
HPLC chromatograms (at 320 nm detection) of aqueous methanolic extracts (25%) of 76 starved individuals of *Bursaridium* sp. (left panel) and bulk dinoflagellates from Oberer Plenderlesee (right panel). We detected the 4 MAAs mycosporine-glycine (MG), shinorine (SH), palythine (PI), and asterina-330 (AS). Note different y-axes.

**Figure 4.**
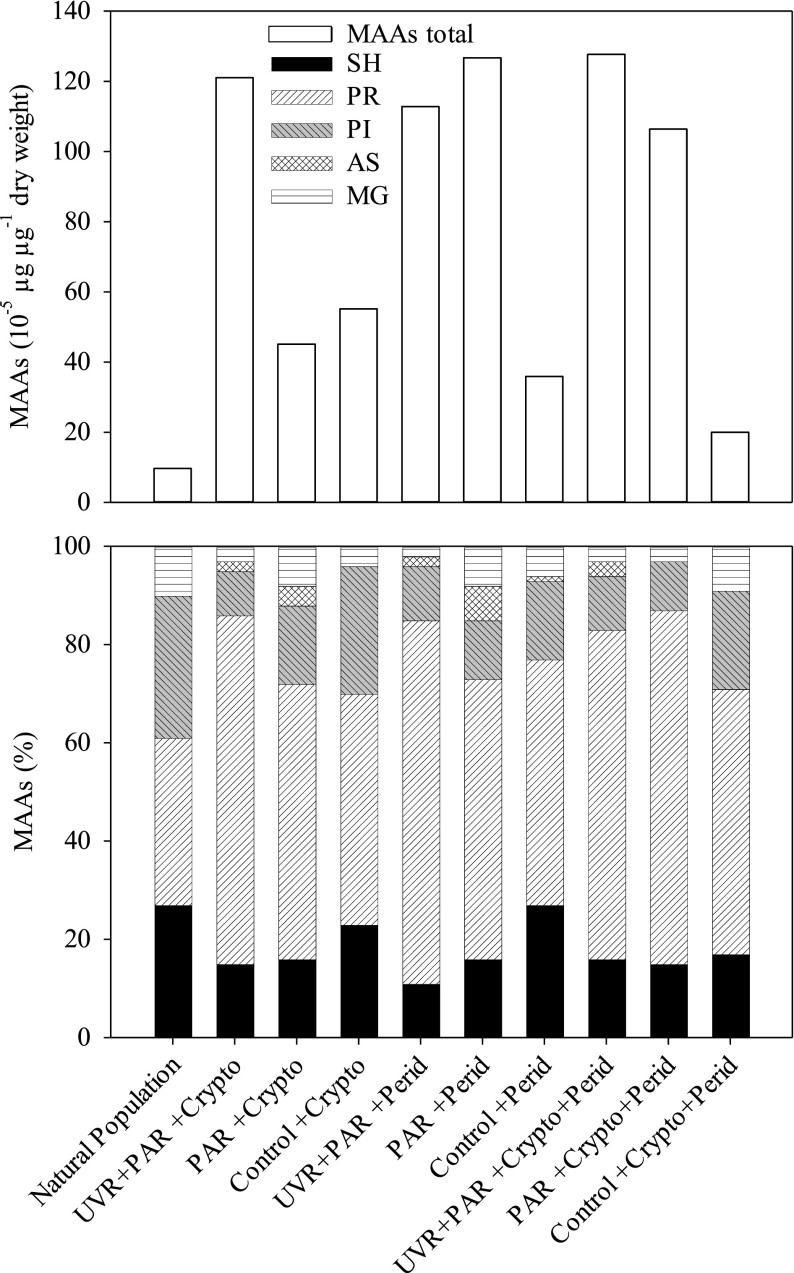
Total MAAs concentrations (10^−5^ μg μg^−1^ DW, upper panel) and their relative contribution (%, lower panel) in a natural *Bursaridium* population from Schwarzsee ob Sölden (SOS) after a feeding experiment in which individuals of *Bursaridium* were fed *Cryptomonas* sp. (MAAs-negative), *Peridinium inconspicuum* (MAA-positive), and a mixture of both. Ciliates were exposed under UVR+PAR, PAR only and a dark control. For details see text.

## *MAAs in* Bursaridium, *lake seston, and dinoflagellates*

We identified the dominant MAAs shinorine (SH), palythine (PI), porphyra-334 (PR), and asterina-330 (AS) in the *Bursaridium* populations from the 3 clear lakes (Fig. 2). In *Bursaridium* from RIF, no MAAs were detected, and only traces of 4 MAAs (i.e., SH, PR, PI, and usujirene [US]) in the seston (0.13 ± 0.1 μg [μg Chl-*a*]^−1^) were found. In HAI, we identified the same 2 MAAs in *Bursaridium* over all sampling years: 89 ± 4% SH and 11 ± 4% AS in concentrations of 33.9 ± 21.0 10^−5^ μg μg^−1^ DW; seston MAAs were detected in concentrations of 20.0 ± 4.5 μg [μg Chl-*a*]^−1^, and proportions of MAAs were 86 ± 0% SH, 13 ± 0% AS, 1 ± 0% PI, <1% mycosporine-glycine (MG), and traces of PR, PI, US, and palythene (PE). In SOS, we identified 7 MAAs in *Bursaridium* over all sampling years: 11 ± 1% MG, 39 ± 16% SH, 15 ± 17% PR, 30 ± 6% PI, 3 ± 4% AS, and 1 ± 2% PE and US, respectively, in total concentrations of 9.0 ± 1.6 10^−5^ μg μg^−1^ DW. In OPL, we identified 4 MAAs in *Bursaridium* in the 2 sampling years, respectively: 4% and 0% MG, 72% and 33% SH, 20% and 9% PI, and 4% PE, in total concentrations of 21.8 and 7.0 10^−5^ μg μg^−1^ DW; the bulk MAAs (MG, SH, PI, AS) detected in the dinoflagellate bloom were the same as analyzed in the individuals of *Bursaridium* (Fig. 3).

### Sensitivity test

No significant mortality was observed among treatments after 6 h of irradiation (*P* = 0.735 in 2011, *P* = 0.815 in 2012).

### Feeding experiment

No significant cell losses of *Bursaridium* were found after both exposure days (*P* < 0.050). The food alga *P. inconspicuum* synthesized 40% SH, 37% MG, 22% AS, and, 1% PI; in *Cryptomonas* sp., no MAAs were detected. Overall, the individual MAAs content per ciliate increased in all approaches: UVR+PAR (112.8–127.7 10^−5^ μg μg^−1^ DW), PAR (45.1–126.7 10^−5^ μg μg^−1^ DW), and controls (20.0–55.2 10^−5^ μg μg^−1^ DW) compared to the natural population (9.7 10^−5^ μg μg^−1^ DW; Fig. 4). Except for PR, which dominated in the relative proportions of all MAAs observed, the MAAs were similar in all treatments (61 ± 10% PR > 17 ± 5% SH > 15 ± 6% PI > 5 ± 3% MG), except for 2 ± 2% AS not detected in the *Cryptomonas* treatment (control) or in the *Peridinium*+*Cryptomonas* treatment (control, and PAR).

## Discussion

Regarding protists and especially ciliates, remote high mountain lakes are still under-sampled habitats (e.g., Foissner 2006). The conspicuous ciliate *Bursaridium* that we isolated from several Austrian alpine lakes occurs at water temperatures <12 °C in low numbers (Table 2, Fig. 1; Fried 1998); however, the species is one of the few ciliate species found in such lakes. To prevent misidentification of the species in future studies, we briefly mention a methodological concern: when the ciliates are preserved without applying a subsequent specific silver staining method revealing the highly characteristic ciliary pattern (e.g., with Lugol’s or mercury chloride fixatives only), the species cannot be differentiated from its congener *Bursaridium pseudobursaria* without investigating living cells (B.S. pers. observ.). Further, disregarding our multitudinous and finally unsuccessful cultivation approaches, we collected each individual *Bursaridium* by hand directly out of the original lake water sample to achieve MAAs analyses and experimental approaches. Irrespective of our difficulties and efforts, we are able to report valuable information on the occurrence, environmental conditions, and the presence of sunscreen compounds for this delicate and prominent protist. Most important, this is the first report on the presence of MAAs in a heterotrophic ciliate species, although several other ciliates such as *Cyclidium* spp., *Glaucoma* sp., *Spirostomum teres*, and *Balanion planctonicum* have been previously tested (Sommaruga and Buma 2000, Sanders et al. 2005, Sonntag et al. 2011b; B.S. unpubl. data).

The composition of the MAAs investigated in the natural *Bursaridium* populations varied depending on a lake’s respective alga or seston (Table 3, Fig. 2). We here show that the ciliates exposed to natural and artificial UVR and PAR regulated their MAA content, including the acquisition from MAAs-rich algal food, assuming an adaptation to thrive under high incident solar radiation conditions (Fig. 3 and 4). A dietary accumulation of MAAs has been investigated for diverse marine and freshwater organisms including copepods, sea urchins, krill, and fish (e.g., Caroll and Shick 1996, Mason et al. 1998, Newman et al. 2000, Moeller et al. 2005, Hylander and Jephson 2010, Orfeo et al. 2011). Moreover, MAA fluctuations in dietary algae were caused by differences in the irradiation spectrum and recovery in the consumer (Antarctic krill; Newman et al. 2000). In another study under PAR exposure, PR increased in the dinoflagellate *Alexandrium tamarense*, and Callone et al. (2006) suggested the existence of a yet unknown process inducing MAAs synthesis after only a few hours.

Elevated PR concentrations under experimental conditions can be explained by the transformation of so-called primary MAAs (MG, SH, and PR) into other MAAs (Shick 2004, Carreto and Carignan 2011). For example, in a cyanobacterium under UV-B exposure, MG was transferred to SH or PR (Portwich and Garcia-Pichel 2003). Because MAA concentrations increased in all our approaches during the feeding experiment, including the controls, we cannot exclude that the ciliates themselves up-regulated their MAAs-content irrespective of the presence of UVR and/or PAR (Fig. 4). The biochemical pathways involved in MAAs synthesis have yet not been identified for ciliates, assuming that these protists received MAAs both from food and/or algal symbionts (Balskus and Walsh 2010, Singh et al. 2010, Osborn et al. 2015). UVR and PAR are not necessarily responsible for an increase in MAAs, however, because some studies showed that under environmental stress conditions, MAAs can have other physiological functions, including osmotic regulation or antioxidant activity of MG, SH, and PR under oxidative stress (Dunlap and Yamamoto 1995, Oren and Gunde-Cimerman 2007, Coba et al. 2008, Singh et al. 2008).

Surface avoidance by daily vertical migration is one well-known photoprotective strategy of zooplankton to escape high solar radiation around midday (e.g., Rautio and Tartarotti 2010). Traveling downward or remaining in depths where UVR wavelengths are more strongly attenuated is also known for other plankton, including ciliates (Sonntag et al. 2011a, Kammerlander et al. 2016, Slaveykova et al. 2016). Lower individual MAAs concentrations in the ciliates from SOS, in contrast to the highest situated and shallower lake HAI (except for 2009), may indicate that *Bursaridium* from SOS escapes high solar irradiation by persisting in greater depths (Table 1 and 3, Fig. 2). In a transplantation experiment in which a ciliate community originating from a less UV transparent lake was exposed at the 10% attenuation depth of 320 nm in a high mountain lake, no significant mortalities were investigated for the dominant species in contrast to surface exposure (Sonntag et al. 2011b).

In conclusion, the *Bursaridium* populations from the transparent alpine lakes observed here were equipped with a set of MAAs and well adapted to potentially damaging UVR levels. This study illustrates that *Bursaridium* from alpine lakes can acquire MAAs from algal food, and evidence indicates that these ciliates may also regulate their MAAs content. We cannot rule out that in our feeding experiment, diverse stress factors including the experimental handling were responsible for an MAA increase in all treatments, but future studies need to address this issue. A physiological adaptation to elevated incident solar radiation, as shown previously for *A. chlorelligera*, implies an ecological advantage not only to survive in UV transparent high mountain lakes, but also to explore food resources over the whole water column. Our results support the hypothesis that MAAs with high absorption efficiencies over a wide range of UVR wavelengths play a major role in minimizing damaging effects of solar UVR on ciliates.

## Funding

This work was supported by Austrian Academy of Science [grant number DOC-fForte 22883], Austrian Science Fund [grant number P16559-B06], [grant number P21013-B03], [grant number I2238-B25].
